# The complete chloroplast genome of *Abutilon theophrasti* medic (Malvaceae)

**DOI:** 10.1080/23802359.2021.1886886

**Published:** 2021-03-16

**Authors:** Yanping Lv, Yuqi Yi, Rongchun Han, Xiaohui Tong, Hiroki Takahashi

**Affiliations:** a School of Pharmacy, Anhui University of Chinese Medicine, Hefei, China; b School of Life Sciences, Anhui University of Chinese Medicine, Hefei, China; c Medical Mycology Research Center, Chiba University, Chiba, Japan

**Keywords:** Chloroplast genome, Malvaceae, *Abutilon theophrasti*

## Abstract

*Abutilon theophrasti* Medic is a traditional Chinese medicine, which can be seen nearly everywhere in China. In order to study its complete chloroplast genome, we collected leaves and obtained chloroplast genome information through next-generation sequencing. It showed that the genome whole length is 160,331 bp, resulted from 24,578,194 raw reads with 3,669,530,829 bases in total, and the GC contents ratio is 36.90%. Besides, the large single-copy region (LSC) is 89,006 bp, the small single-copy region (SSC) 20,149 bp, and inverted repeat (IR) 25,588 bp. The chloroplast genome encodes 76 genes, which contains 38 protein genes, five rRNA genes, and 33 tRNA. By conducting phylogenetic analysis for *A.theophrasti*, plants from genus *Gossypium* demonstrated close relationship with it.

In ancient China, *Abutilon theophrasti* Medic, as a medicinal plant that was easy to get, was frequently utilized to evacuate the heat and extra water in patients’ body (Tian et al. 2019). But because of the decreasing medicine resources, *A. theophrasti* was also plant and bred in some areas in China nowadays (Wu et al. 2018). With the advancement of medical and research technology, it was found that *A. theophrasti* was good at insect control and weeding (Cao et al. 2017). So it was widely used in the agricultural field recently (Alms et al. 2016). Abundant studies have been conducted regarding its chemical components, pharmacological actions (Wu et al. 2018), as well as the gene analysis and gene expression (Mamadalieva et al. 2014). However, information on its chloroplast genome is still unclear. To address this, we extracted the chloroplast genomic DNA and after sequencing, the features of the chloroplast genome were annotated. It will provide valuable information for further research on this plant.

The fresh leaves were sampled from Hefei, Anhui Province, China (N31°56′32″; E117°23′18″). The specimen was deposited in Herbarium of Anhui University of Chinese Medicine with the voucher number 200713AH002. Next-generation sequencing was carried out by Genewiz Co. Ltd. (Suzhou, China). Fragments of chloroplast genomic DNA were first extracted from the fresh leaves. After repairing the ends and ligating indexed paired-end adapters, the sequencing information was retrieved. Once we obtained the raw sequence data, followed by quality control and assembly with velvet software (Zerbino and Birney 2008), contigs were gapfilled by SSPACE (Boetzer et al. 2011). Subsequently, gene-finding tools (prodigal, V2.6.3) and public databases were utilized for annotation. For NR annotation, DIAMOND (version 0.8.15) was applied and for KEGG database, BLAST software (version 2.2.28+) was used (Buchfink et al. 2015).

By analyzing the exact sequence of the chloroplast genome of *A. theophrasti*, we found its full length is 160,331 bp, assembly from 24,578,194 reads (3,669,530,829 bases), and the GC ratio is 36.9%. What’s more, it contains 76 genes in all, including 38 protein genes, five rRNA, and 33 tRNA genes. The large single-copy region (LSC) is 89,006 bp long, and the small single-copy region (SSC) is 20,149 bp, with inverted repeat (IR) as 25,588 bp.

Available chloroplast genomic information from genus *Abutilon* was limited. We selected 10 relevant plant species to study their phylogenetic position with *A. theophrasti*. Alignment and a maximum likelihood (ML) tree were concluded by using MEGA X, with the combined bootstrap method (1000 replicates). The Tamura-Nei substitution model was used in the ML analysis (Kumar et al. 2018). According to the phylogenetic tree, it was found that plants from genus *Gossypium* showed closer relationship with *A. theophrasti* (underlined) compared with species from other taxonomic groups (Figure 1).

**Figure 1.**
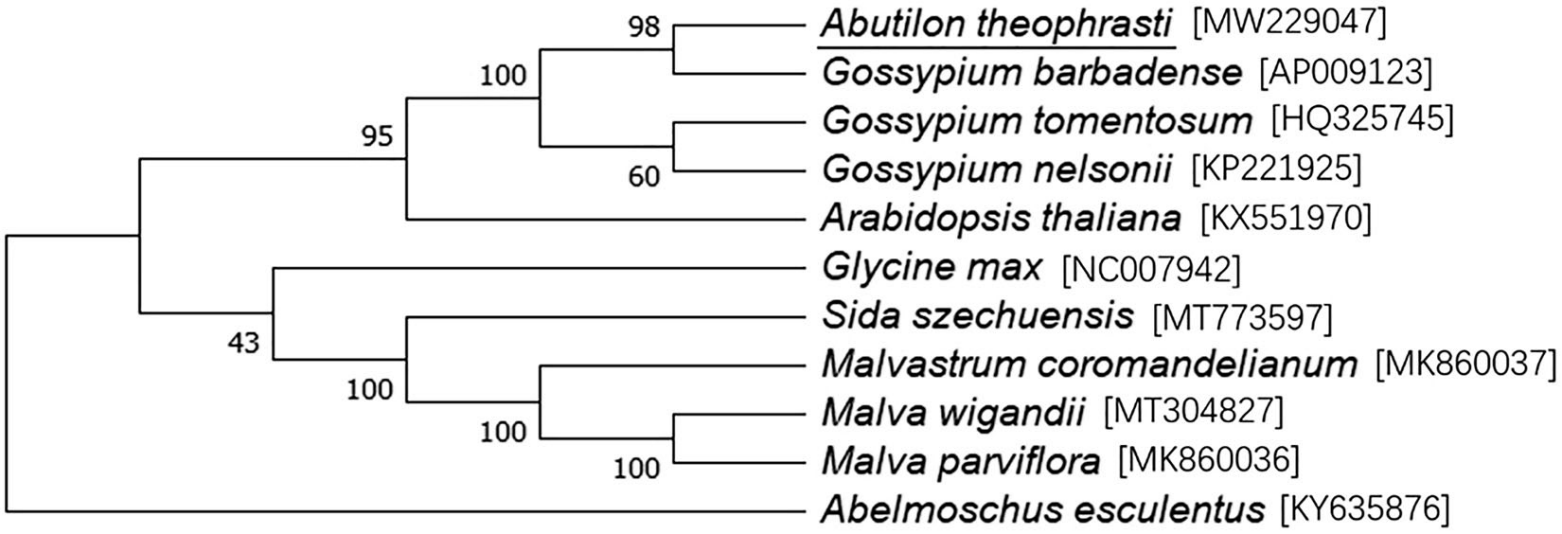
Maximum likelihood phylogenetic tree based on the chloroplast genome sequences from 11 related species. Values along branches refer to the percentage of replicate trees where the associated taxa clustered together.

## Data Availability

The genome sequence data that support the findings of this study are openly available in GenBank of NCBI at https://www.ncbi.nlm.nih.gov under the accession number MW229047. The associated BioProject, SRA, and Bio-Sample numbers are PRJNA684955, SRR13249439, and SAMN17073185 respectively.
